# *In Silico*, *In Vitro* and *In Vivo* Pharmacodynamic Characterization of Novel Analgesic Drug Candidate Somatostatin SST_4_ Receptor Agonists

**DOI:** 10.3389/fphar.2020.601887

**Published:** 2021-01-27

**Authors:** Boglárka Kántás, Éva Szőke, Rita Börzsei, Péter Bánhegyi, Junaid Asghar, Lina Hudhud, Anita Steib, Ágnes Hunyady, Ádám Horváth, Angéla Kecskés, Éva Borbély, Csaba Hetényi, Gábor Pethő, Erika Pintér, Zsuzsanna Helyes

**Affiliations:** ^1^ Department of Pharmacology and Pharmacotherapy, Medical School, University of Pécs, Pécs, Hungary; ^2^ János Szentágothai Research Center and Center for Neuroscience, University of Pécs, Pécs, Hungary; ^3^ PharmInVivo Ltd., Pécs, Hungary; ^4^ Department of Pharmacology, Faculty of Pharmacy, University of Pécs, Pécs, Hungary; ^5^ Avicor Ltd., Budapest, Hungary; ^6^ Gomal Centre of Pharmaceutical Sciences, Gomal University, Khyber Pakhtoonkhwa, Pakistan; ^7^ Algonist Biotechnolgies GmbH, Vienna, Austria

**Keywords:** neuropathic pain, drug discovery, G protein coupled receptor, somatostatin, somatostatin receptor subtype 4, molecular, modeling

## Abstract

**Background:** Somatostatin released from the capsaicin-sensitive sensory nerves mediates analgesic and anti-inflammatory effects via its receptor subtype 4 (SST_4_) without influencing endocrine functions. Therefore, SST_4_ is considered to be a novel target for drug development in pain, especially chronic neuropathy which is a great unmet medical need.

**Purpose and Experimental Approach:** Here, we examined the *in silico* binding, SST_4_-linked G protein activation and β-arrestin activation on stable SST_4_ expressing cells and the effects of our novel pyrrolo-pyrimidine molecules (20, 100, 500, 1,000, 2,000 µg·kg^−1^) on partial sciatic nerve ligation-induced traumatic mononeuropathic pain model in mice.

**Key Results:** The novel compounds bind to the high affinity binding site of SST_4_ the receptor and activate the G protein. However, unlike the reference SST_4_ agonists NNC 26-9100 and J-2156, they do not induce β-arrestin activation responsible for receptor desensitization and internalization upon chronic use. They exert 65–80% maximal anti-hyperalgesic effects in the neuropathy model 1 h after a single oral administration of 100–500 µg·kg^−1^ doses.

**Conclusion and Implications:** The novel orally active compounds show potent and effective SST_4_ receptor agonism *in vitro* and *in vivo*. All four novel ligands proved to be full agonists based on G protein activation, but failed to recruit β-arrestin. Based on their potent antinociceptive effect in the neuropathic pain model following a single oral administration, they are promising candidates for drug development.

## Introduction

Targeting somatostatin receptors as novel analgesic and anti-inflammatory drug developmental approaches has emerged after our team discovered that somatostatin was released from the activated capsaicin-sensitive peptidergic sensory nerve endings into the systemic circulation which leads to anti-inflammatory and anti-hyperalgesic actions at distant parts of the body (Pintér et al., 2006; Szolcsanyi et al., 2011; Pintér et al., 2014; Schuelert et al., 2015; Shenoy et al., 2018; Hernández et al., 2020; Kuo et al., 2020). These effects were mimicked by a synthetic heptapeptide agonist, TT-232, acting on the somatostatin receptors subtype 4 and 1 (SST_4_ and SST_1_) located on both primary sensory neurons and immune cells (Pintér et al., 2002; Helyes et al., 2004; Szolcsányi et al., 2004). J-2156, a highly selective and efficacious non-peptide SST_4_ receptor agonist inhibited nocifensive behavior in the second phase of the formalin test, adjuvant-evoked chronic inflammatory mechanical allodynia, and sciatic nerve ligation-induced neuropathic mechanical hyperalgesia (Sándor et al., 2006). Furthermore, J-2156 decreased neuropeptide release from the peripheral terminals of peptidergic sensory neurones, as well as neurogenic and non-neurogenic acute inflammatory processes and adjuvant-induced chronic arthritic changes (Helyes et al., 2006; Elekes et al., 2008). In accordance with the above findings, in SST_4_ receptor knockout mice acute and chronic inflammatory as well as neuropathic hyperalgesia were more severe than in wild types (Helyes et al., 2009). In addition to the peripheral nervous system, the SST_4_ receptor is present in several central nervous system regions involved in the regulation in pain, such as the spinal cord, hippocampus and amygdala (Schreff et al., 2000; Selmer et al., 2000a; Selmer et al., 2000b). All these data provide strong proof of concept that small molecule non-peptide SST_4_ receptor agonists are promising drug candidates for novel analgesic development. Furthermore, it is also important, that SST_4_ does not mediate endocrine actions of somatostatin.

Based on these data, SST_4_ agonists have recently become the focus of interest and development pipeline of several pharmaceutical companies for the treatment of chronic pain with one compound being tested in phase 1 clinical trial (Lilly, 2020; Stevens et al., 2020). We synthesized and patented novel pyrrolo-pyrimidine molecules (Compound 1, Compound 2, Compound 3, Compound 4) (see details in the Supplementary Materials) (Szolcsányi et al., 2019), and in the present paper we characterize their *in silico* binding, *in vitro* receptor activation and *in vivo* anti-hyperalgesic effects after single oral administration.

## Methods

### *In Silico* Modeling Studies

#### Preparation of Ligand and Target Structures

Five ligand structures were built in Maestro (Schrödinger, 2017). The semi-empirical quantum chemistry program package MOPAC (Stewart, 2016) was used to minimize the raw structures with a PM7 parametrization (Stewart, 2013). The gradient norm was set to 0.001. Force calculations were applied on the energy minimized structures and the force constant matrices were positive definite. The energy-minimized structures were forwarded to docking calculations. The structure of SST_4_ receptor was created by homology modeling using the active form of adrenergic β2-receptor (PDB code: 3p0g) as template. The sequence alignment was performed as in the model constructed and described by Liu and co-workers (Liu et al., 2012). Five homology models generated by Modeller program package (Stewart, 2016) were ranked related to their Discrete Optimized Protein Energy score (DOPE score) value. The first ranked model was energy-minimized and equilibrated by GROMACS 5.0.2 (Abraham et al., 2015) as described in the previous study (Liu et al., 2012). The energy-minimized receptor structure was used as a target in the docking calculations.

#### Docking

Docking of all ligands was performed by AutoDock 4.2.6 (Morris et al., 2009) focused on the extracellular region of the SST_4_ target. In order to reduce false positive conformations, the transmembrane and intracellular target regions were not included in the docking search. Gasteiger-Marsilli partial charges were assigned to both the ligand and target atoms in AutoDock Tools (Morris et al., 2009), and united atom representation was applied for non-polar moieties. Flexibility was allowed at all active torsions of the ligand, but the target was treated rigidly. The grid maps were prepared by AutoGrid 4. The number of grid points was determined by Eq. 1, where L_max_ is the length of the longest ligand structure and x is the number of grid points.

$${\text{L}}_{\max}+5=0.375\text{x}\text{.}$$ (1)

The docking box was centered on the extracellular region of SST_4_ including 66 × 66 × 66 grid points at a 0.375 Å spacing. Lamarckian genetic algorithm was used for global search. After 10 docking runs, ligand conformations were ranked according to the corresponding calculated interaction energy values and subsequently clustered using a root mean square deviation (RMSD) tolerance of 3.5 Å between cluster members. Rank 1 was analyzed and selected as representative structure for each ligand.

### G Protein Activation Assay

Membrane fractions prepared from Chinese hamster ovary (CHO) cells stably expressing the SST_4_ receptor (in Tris-Ethylene glycol bis(2-aminoethyl)tetraacetic acid (Tris–EGTA) buffer (50 mM Tris–HCl, 1 mM EGTA, 3 mM MgCl_2_, 100 mM NaCl, pH 7.4, 10 μg of protein/sample) were used for the investigations. The SSTR4 coding sequence was cloned into a pWPTS-derived lentiviral transfer vector containing an internal ribosomal entry site (IRES) and the green fluorescent protein (GFP) gene. The SSTR4-IRES-GFP construct was driven by the EF1 promoter. HEK293 cells were used to produce the lentiviral particles, by cotransfecting the cells with the SST4 receptor coding “transfer,” pMD.G “helper” and R8.91 “packaging” vectors. The culture media of HEK293 cells containing the lentiviral particles were transferred to the CHO-K1 cells. The virus particles stably transfected the CHO cells creating the stable SSTR4 expressing CHO cell line, which was used in the further experiments. Cell culture media containing the virus particles were transferred onto CHO-K1 cells. These fractions were incubated for 60 min at 30°C in the buffer containing 0.05 nM guanosine triphosphate (GTP), labeled on the gamma phosphate group with ^35^S ([^35^S]GTPγS) and increasing concentrations (0.1 nM–10 µM) of test compounds. 30 μM guanosine diphosphate (GDP) was added in a final volume of 500 µl. We determined the non-specific binding in the presence of 10 μM unlabelled GTPγS and total binding in the absence of test compounds. At the end of the experiment we filtered the samples through Whatman GF/B glass fiber filters using 48-well Slot Blot Manifold from Cleaver Scientific. Filters were washed with ice-cold 50 mM Tris–HCl buffer (pH 7.4) and radioactivity was measured in a β-counter (PerkinElmer Inc., Waltham, MA, United States). Test compound-induced G protein activation was given as percentage of the specific [^35^S]GTPγS binding detected in the absence of agonists (Markovics et al., 2012).

### β-Arrestin Activation Assay

In the PathHunter™ Enzyme Fragment complementation assay (DiscoverX, Fremont, CA), pCMV Mammalian cloning vector is used to drive the CHO-K1 SSTR4 cell lines to express both GPCR fused to a small enzyme donor fragment ProLink (PK), and β-Arrestin tagged with Enzyme Acceptor fragment. Upon stimulation of GPCR, β-arrestin binds to the prolink leading to the complementation of the enzyme fragments. The signal is then detected by adding the chemiluminescent reagent.

β-arrestin2 CHO-K1 SSTR4 cells were plated at a density of 20,000 cells/well in white 96 well plates and incubated overnight at 37°C. Cells were then loaded with a range of SST_4_ receptor agonists’ concentrations (10^−12^–10^−5^ M) in the assay media for 90 min at 37°C. Determinations were made in duplicates. The detection reagents were added and the incubation continued at room temperature for 60 min. The agonist mediated β-arrestin 2 interaction was determined using the detection reagents according to the manufacturer’s instructions. Chemiluminescence indicated as relative luminescence units (RLUs) was measured on EnSpire Alpha Plate Reader (Perkin Elmer).

### Animals and Ethics

Male NMRI (named after the U.S. Naval Medical Research Institute) mice (8–12-week-old, 35–40 g weight) were used in the pain experiments. They have the highest nociceptive threshold among all mouse strains (Leo et al., 2008). Partial sciatic nerve ligation is a well-known, widely used, reproducible method to induce neuropathic pain in mice, characterized by significant allodynia and hyperalgesia, mimicking human neuropathic pain (Malmberg and Basbaum, 1998; Shields et al., 2003). We performed the first series of behavioral experiments with NMRI mice. Since we observed that the individual results show significant differences within each group including the control group, we used male C57Bl/6 mice (12–16-week-old, 25–30 g weight) for this purpose to be comparable with previous behavioral studies (Scheich et al., 2016; Scheich et al., 2017).

Mice were bred in the Laboratory Animal House of the Department of Pharmacology and Pharmacotherapy of the University of Pécs, kept in standard plastic cages at 24–25°C, under a 12–12 h light–dark cycle and provided with standard rodent chow and water *ad libitum*.

The study was designed and conducted according to European legislation (Directive 2010/63/EU) and Hungarian Government regulation (40/2013., II. 14.) on the protection of animals used for scientific purposes. The project was approved by the Animal Welfare Committee of the University of Pécs and the National Scientific Ethical Committee on Animal Experimentation of Hungary and licensed by the Government Office of Baranya County (license No. BA1/35/55-50/2017). We made all efforts to minimize the number and suffering of the animals used in this study. The group size in our experiments was chosen based upon free available power analysis program (Power and Sample Size.com, 2020) and our previous experiences using similar experimental protocols. The minimal required number for sufficient statistical power was 7. After the experiments, mice were sacrificed by cervical dislocation.

### Measurement of the Mechanonociceptive Threshold of the Hindpaw and Partial Sciatic Nerve Ligation-Induced Neuropathic Pain Model of the Mouse

To measure the mechanical threshold of both hindpaws, mice were placed individually in small cages with a framed metal mesh floor. The mechanonociceptive thresholds of the mouse hindpaw were determined with the Dynamic Plantar Aesthesiometer (Ugo Basile Dynamic Plantar Aesthesiometer 37400; Comerio, Italy). This electronic von Frey device applied pressure to the plantar surface of the hindpaw with a blunt-end needle which continuously rose for 4 s until 10 g force. The force at which a paw withdrawal response occurred is registered by the equipment and it was taken as the mechanonociceptive threshold. Paw withdrawal automatically turned off the stimulus.

After conditioning, three control mechanonociceptive hindpaw threshold measurements were performed on three consecutive days. Mice were then anesthetized by the combination of ketamine (100 mg·kg^−1^, i.p.) and xylazine (10 mg·kg^−1^, i.p.) and placed under a dissection microscope. The right sciatic nerve was isolated from the surrounding connective tissues at a proximal site and the dorsal 1/3–1/2 of the nerve was tightly ligated with only one 8–0 silk suture in order to induce traumatic sensory mononeuropathy (Seltzer et al., 1990). The surgery was performed under aseptic conditions, including sterile gloves, mask and sterile instruments. The animals were placed on a heating plate after the operation and monitored until complete awakening. The mechanonociceptive threshold of the plantar surface of the hindpaws was measured again on the seventh postoperative day in order to detect the development of the neuropathic pain-like state mechanical hyperalgesia in response to the nerve ligation expressed as percentage decrease compared to the mean three initial (pre-surgery) control values. Animals that failed to show at least 20% hyperalgesia were excluded from the experiment (107 out of 358 animals; 70% success rate of the operation), since they did not have obvious neuropathic pain. Subsequently, the test compounds or the vehicle methylcellulose were applied orally (in a volume of 20 ml·kg^−1^ body weight) and threshold measurements were repeated 60 min later in order to compare mechanical hyperalgesia before and after the treatment. The anti-hyperalgesic effects of the test compounds were expressed in percentage by the following formula: ((hyperalgesia before drug treatment—hyperalgesia after drug treatment)/hyperalgesia before drug treatment) · 100. The intact contralateral paws were also measured for comparison.

The experiment consisted of 15 separate series and the animals were randomized to receive the respective treatment or the vehicle. The experimenter was blinded from the treatment the animals received. The number of animals in the control group was at least four per day to minimize the bias caused by the different experimental days. Therefore, the total number of animals in the different experimental groups ranged from 7 to 19 (see details in the respective figures).

### Determination of Anxiety and Spontaneous Locomotor Activity: Elevated Plus Maze (EPM) and Open Field Test (OFT)

Anxiety behavior was examined in the EPM apparatus consisting of two open and two closed arms that are extended from a common central platform. The platform was 60 cm above floor level, the floor and the walls of each arm were plastic and painted gray. Sixty min after oral administration of the vehicle or Compound 2 (500 µg·kg^−1^), mice were placed in the center of the maze and the time they spent in the open arms during the 5-min experiment was measured (Lister, 1987; Kraeuter et al., 2019a; He et al., 2020). The surface of the maze was cleaned with 70% ethyl alcohol after each test to remove permeated odors from previous animals. There were 10 mice in each group.

Spontaneous locomotor activity and anxiety level was determined in the OFT composed of a plastic box (39 cm × 39 cm × 39 cm) with white floor and gray walls. Sixty min after the oral administration of the vehicle or Compound 2 (500 µg·kg^−1^), mice were placed individually in the center of the box and were observed for 5 min. The arena was cleaned with 70% ethyl alcohol after each trial to remove permeated odors from previous animals (Kraeuter et al., 2019b; He et al., 2020). Behavioral parameters were recorded and analyzed by EthoVision XT 8.0 (Noldus Information Technology, Wageningen, Netherlands) motion tracking software. The number of the animals are 10 in each group.

### Data and Statistical Analysis

Graphs and calculations were made using GraphPad Prism (GraphPad Prism version 8.0.1 for Windows). Curves of both G protein activation and β-arrestin 2 recruitment assays were fit by nonlinear regression using the sigmoidal dose–response equation. In the G protein activation assay we performed three experiments in triplicates. In β-arrestin assay, the experiments were conducted twice. In one experiment there were six different concentrations of each drug, each concentration was tested in duplicates to provide *n* = 2.

Results are expressed as means ± S.E.M. The maximum responses for all compounds in β-arrestin 2 recruitment assay were compared using one-way ANOVA with Dunnett’s post hoc test. Data of neuropathic pain model were analyzed by one-way ANOVA Bonferroni's Multiple Comparison Test for comparing the anti-hyperalgesic effects in the different groups. Data of behavioral experiments were compared using Student's unpaired t‐test except the number of rearings which were made using the Mann‐Whitney U‐test. The levels for statistically significant differences were set as *p < 0.05, **p < 0.01.

### Materials

In the SST_4_ receptor activation assay all the compounds were dissolved in dimethyl sulfoxide (DMSO). The concentration of the stock solutions was 10 mM, that was diluted with distilled water or assay medium to reach the final concentrations. For the *in vivo* experiments 1 mg of the compounds was suspended thoroughly in 1 ml 1.25% methylcellulose solution dissolved in sterile bidistilled water to get a 1,000 µg·ml^−1^ stock solution freshly every experimental day. Most microsuspensions looked opalescent, they were shaken properly, sonicated, and further diluted with 1.25% methylcellulose to obtain the 1, 5, 25, 50 and 100 µg·ml^−1^ solution for oral administrations (20 ml·kg^−1^ body weight for the 20, 100, 500, 1,000 and 2,000 µg·kg^−1^ dose). The solutions were shaken and sonicated again directly before use. The vehicle was always 1.25% methylcellulose dissolved in sterile bidistilled water.

Tris–HCl (PubChem CID: 93573), EGTA (PubChem CID: 6207), MgCl_2_ (PubChem CID: 5360315), NaCl (PubChem CID: 5234): Reanal, Budapest, Hungary; GTP (PubChem CID: 135398633) : BioChemica International Inc., Melbourne, FL, United States; GDP (PubChem CID: 135398619), urea (PubChem CID: 1176), acetic acid (PubChem CID: 176): Sigma, St. Louis, MO, United States; dimethyl sulfoxide (DMSO, PubChem CID: 679): Szkarabeusz Ltd., Pécs, Hungary; [^35^S]GTPγS: Institute of Isotopes, Budapest, Hungary; CHO-K1 cells: European Collection of Authenticated Cell Cultures (ECACC Cat# 85051005, RRID:CVCL_0214), SST_4_ receptor-expressing cell line was prepared in our laboratory; methylcellulose (MC; Ph. Eur. V.; PubChem CID: 44263857): Central Pharmacy of the University of Pécs, Pécs, Hungary; hsstr_4_ cAMP CHO-K1 (RRID:CVCL_KV83) and hsstr_4_ β-arrestin 2 CHO-K1 cells (RRID:CVCL_KZ14): DiscoverX, Fremont, CA; methylcellulose (MC;Ph.Eur.V.; PubChem CID: 44263857): Central Pharmacy of the University of Pécs, Pécs, Hungary.

## Results

### *In Silico* Modeling and Binding Assay

**FIGURE 1:**
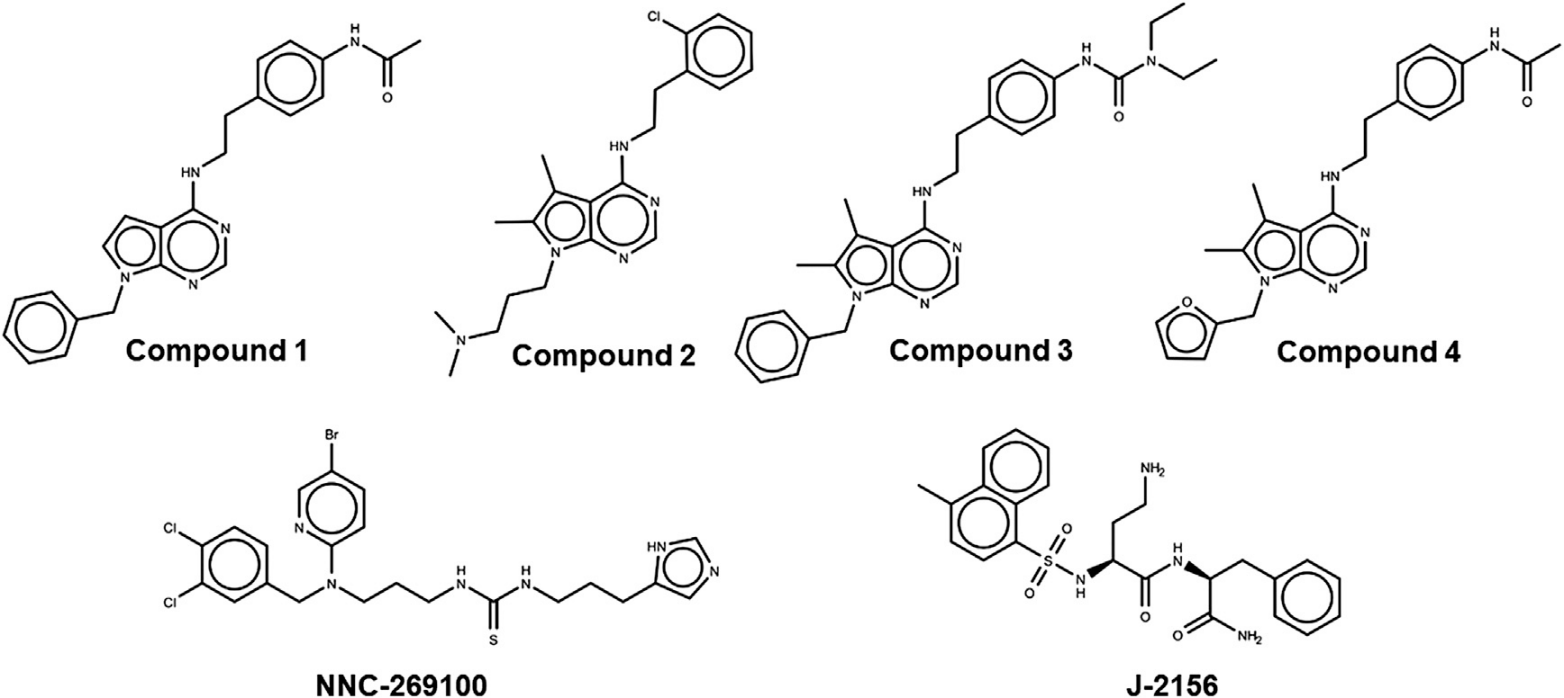
Lewis structures of the tested new pyrrolo-pyrimidine ligands (upper panel) and the high affinity reference molecules (lower panel).

Two high affinity SST_4_ agonist reference compounds NNC-269100 and J-2156 (Liu et al., 1998) were used in the present study. They were shown to bind a region called high affinity binding pocket in previous studies composed of amino acids Tyr18, Val67, Ser70, Ala71, Cys83, Asp90, His258, Val259, Ile262, Leu263. Serial numbering of target residues follows that of the previous study (Liu et al., 2012). Docking of the references and four new Compounds (Figure 1) to the SST_4_ target was performed as described in Methods. It was found that interaction energy values of the docked representatives of Compounds 1–4 do not show significant differences if compared with the high affinity reference molecules NNC-269100 and J-2156 (Table 1) Amino acids interacting with the representative docked ligands are marked with a cross in Table 1. Reference molecules have interaction with eleven target (showed with gray color in Table 1) residues that are identical (10^−5^ M) more than 60% (showed with double cross in Table 1). Fit % means the ratio of identical interacting residues of the references calculated for each compound. It shows that the ratio of interacting target residues for Compounds 1–3 is similar as that of the reference molecules. However, fit % of Compound 4 is 45%, it has interaction with Asp90, the key amino acid suggested essential role in ligand binding and receptor activation. Analyzes of the residues interacting with the representative docked ligand conformations within 3.5 Å showed that the new compounds maintain the contact with amino acids similarly to reference molecules, likewise to the interaction energy. Thus, reference ligands and new compounds have overlapping binding site on SST_4_.

**TABLE 1:** Target residues interacting with representative docked ligand structures within 3.5 Å.

	NNC-269100	J2156	Compound 1	Compound 2	Compound 3	Compound 4
Tyr18			x	x	x	
Val67	X		x	x	x	
Ser70	Xx	xx	xx	xx	xx	
Ala71				x		
Trp76	X		x	x	x	
Cys83	Xx	xx	xx		xx	xx
Arg84			x			x
Val86					x	
Leu87	Xx	xx	xx	xx	xx	
Asp90		x		x	x	x
Pro153			x			x
Asn163			x			x
Pro169	xx	xx	xx	xx	xx	xx
Ala170			x			x
Trp171	xx	xx	xx		xx	xx
His258	xx	xx				xx
Val259		x		X		
Ile262	xx	xx	xx	xx	xx	
Tyr265			X			
Fit (%)	82	82	73	73	82	45
E_inter_	−6.58	−6.58	−8.17	−6.67	−7.97	−7.17

Amino acids interacting with the docked representatives within 3.5 Å are marked with a cross. Gray color shows the amino acids interacting with reference molecules. Double cross indicates amino acids interacting with both reference molecules.

**FIGURE 2:**
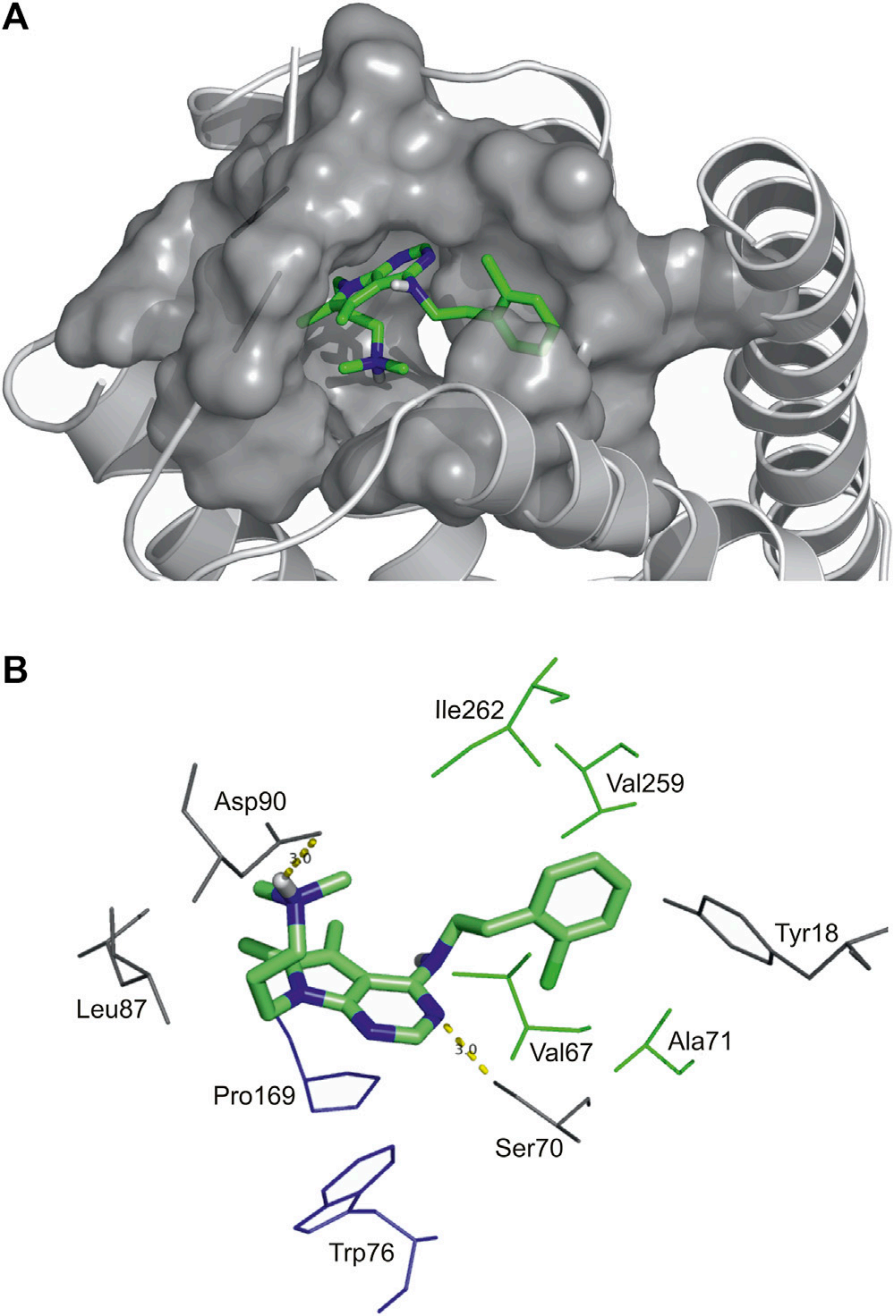
High affinity binding pocket with Compound 2 in SST_4_ receptor **(A)**, Binding pattern of Compound 2: hydrophobic pocket composed of Val67, Ala71 in TM2, Val259 and Ile262 in TM7, aromatic-aromatic interactions with Pro169 and Trp76, H-bonds (yellow) with Asp90 and Ser70 **(B)**.

As an example, atomic details of binding of Compound 2 to SST_4_ is further shown in a close-up view (Figure 2). The chlorobenzyl group of Compound 2 is buried in a hydrophobic pocket formed by Val67, Ala71 in TM2, Val259 and Ile262 in TM7 (TM2/TM7 hydrophobic cavity (Liu et al., 2012). The 7H-pyrrolo[2,3-d]pyrimidine core of molecule has aromatic-aromatic interactions with Pro169 and Trp76 and stabilized by a H-bond with Ser70. Furthermore, there is an ionic interaction between Asp90 on TM3 and tertiary amine group of Compound 2. It is presumed based on site-directed mutagenesis studies that an ionic interaction between Lys9 of endogenous peptide and the conserved aspartic acid on TM3 of all SST receptors has a crucial role in ligand binding and receptor activation (Kaupmann et al., 1995; Nehrung et al., 1995; Ozenberger and Hadcock, 1995; Chen et al., 1999; Liu et al., 2012).

### SST_4_ Receptor-Coupled G Protein Activation

**FIGURE 3:**
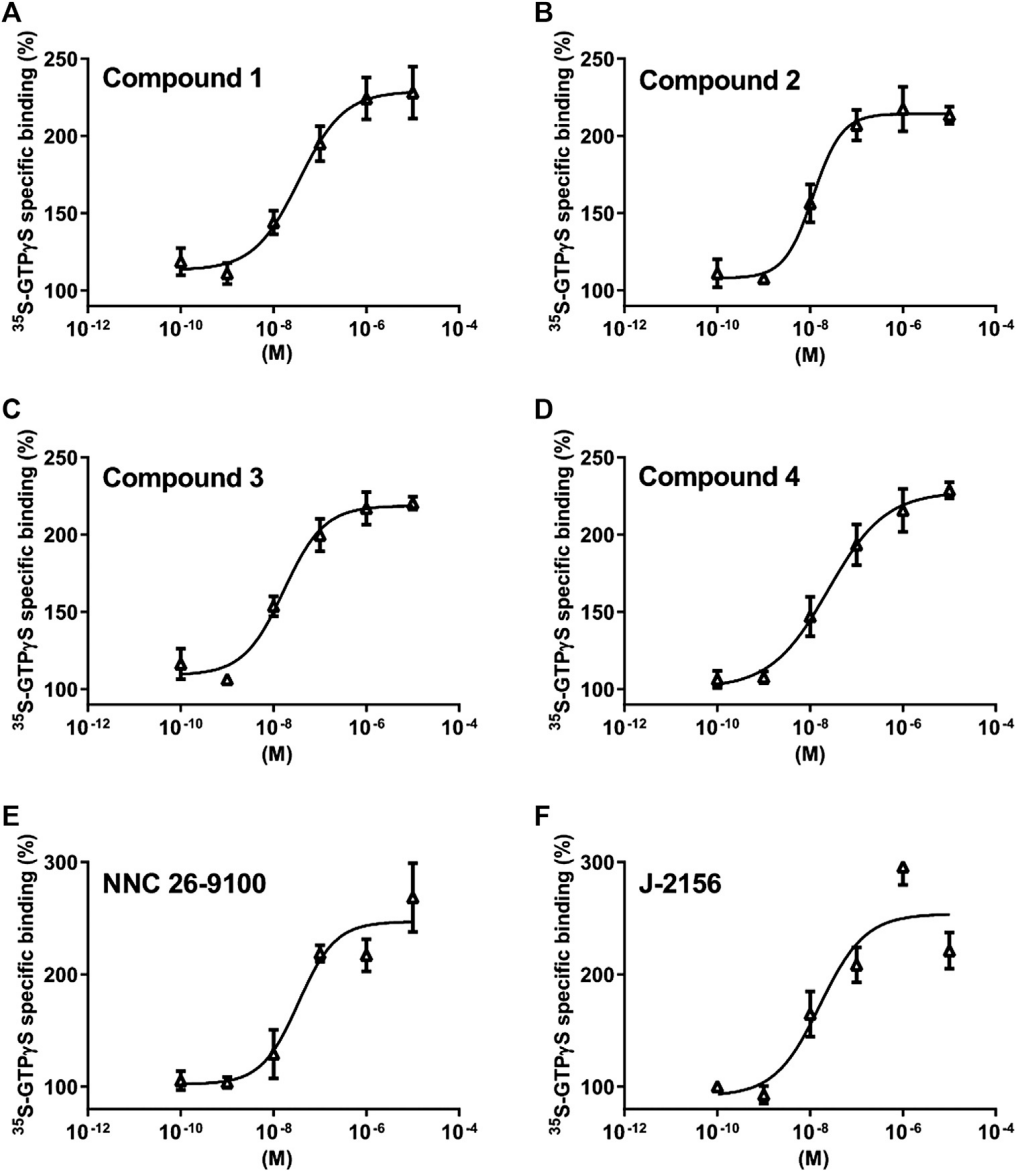
Effect of Compounds 1–4 compared with reference molecules NNC 26-9100 and J-2156 on SST_4_ receptor-linked G protein activation. [^35^S]GTPγS binding induced by the compound in SST_4_-expressing CHO cells. The ligand-stimulated [^35^S]GTPγS binding reflects the GDP–GTP exchange reaction on α subunits of G proteins by receptor agonists. Increasing concentrations of all compounds result in similar concentration-dependent stimulations of [^35^S]GTPγS binding. Each data point represents the mean ± SEM of *n* = 3 independent experiments, each performed in triplicates.

Based on the *in silico* binding results, the SST_4_ receptor activating potential of the new compounds was measured and compared to the reference agonist NNC 26-9100 and J-2156 (Engström et al., 2005). We found concentration-dependent stimulation in the [^35^S]GTPγS binding assay on SST_4_-expressing CHO cells (Figure 3.). The EC_50_ values demonstrating the potency of the ligands were, 75 , 28 , 16 and 24 nM for Compound 1, 2, 3 and 4, respectively (n=3 independent experiments with each compound). The maximal activation values over the basal activities of the receptor showing the efficacy of the compounds were 242.7 ± 26%, 213 ± 9%, 220 ± 7% and 228.7 ± 9%, in cases of Compounds 1, 2, 3 and 4, respectively. Thus, all these compounds are potent and effective SST_4_ receptor agonists.

### Effects of Compounds 1–4 on SST_4_ Activation-Related β-Arrestin 2 Recruitment

**FIGURE 4:**
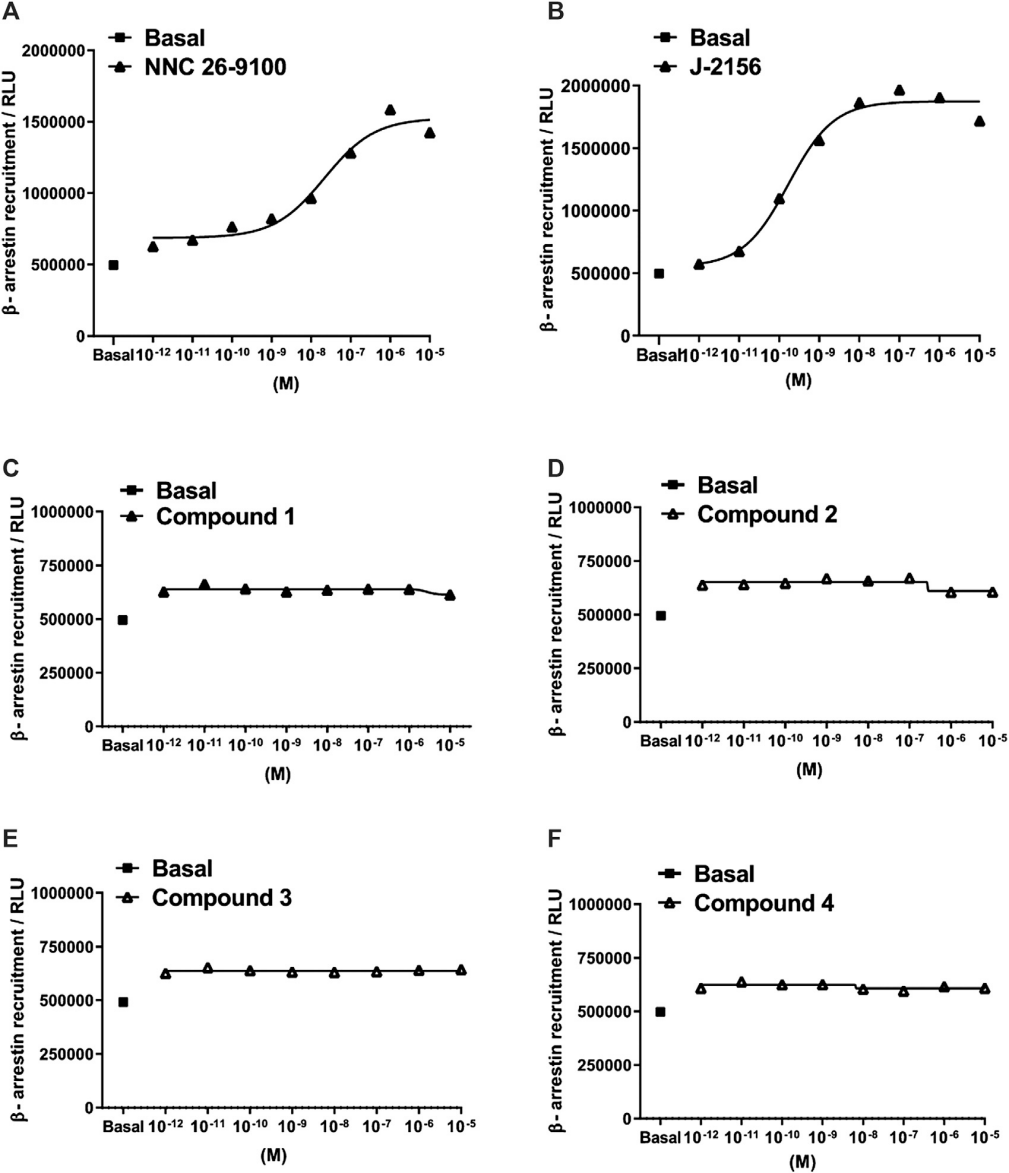
Concentration-response curves of Compounds 1–4 in the β-arrestin 2 recruitment assay. Data represent concentration–response curves of the novel compounds expressed as relative luminescence units (RLU) in comparison to the reference compounds NNC 26-9100 and J-2156. All values are means ± SEM (*n* = 2 experiments). In each experiment, data points were obtained in duplicates.

Subsequently, we investigated the ability of the agonists to mediate β-arrestin two recruitment, measured as an increase in the chemiluminescent signal. The novel ligands displayed no detectable β-arrestin 2 recruitment in the PathHunter assay (testing range: 10^−12^–10^−5^ M). However, the reference compounds, NNC 26-9100 and J-2156 showed marked β-arrestin 2 recruitment (Figure 4).

### Anti-Hyperalgesic Effect of Compounds 1–4 in the Partial Sciatic Nerve Ligation-Induced Neuropathy Model

**FIGURE 5:**
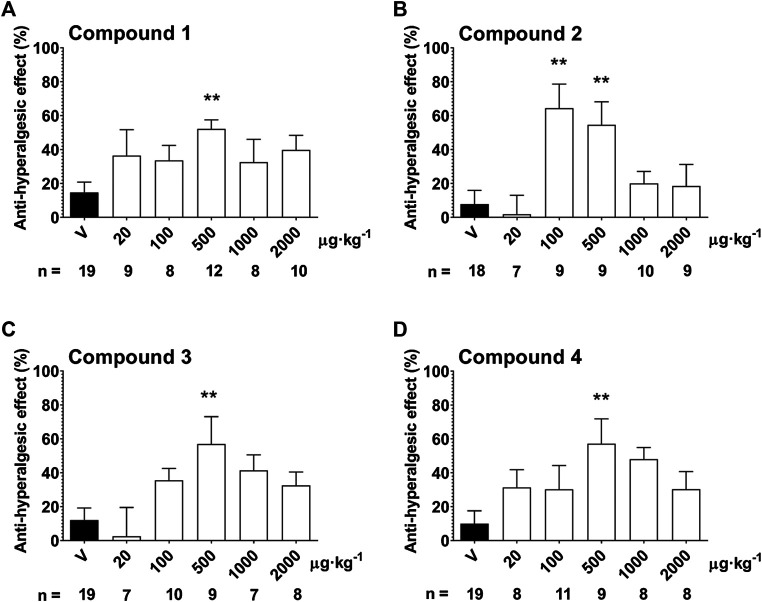
Anti-hyperalgesic effect of a single oral treatment with Compounds 1–4 7 days after partial tight ligation of the sciatic nerve in the mouse. Columns represent anti-hyperalgesic effect 60 min after treatment with the respective test compound compared to pre-treatment control values. Each column represents the mean + S.E.M. of *n*. Data were analyzed with one-way ANOVA Bonferroni’s Multiple Comparison Test (*p < 0.05, **p < 0.001 vs. vehicle control values).

In response to the partial sciatic nerve ligation, 37.3 ± 0.8% mechanical hyperalgesia (drop of the mechanonociceptive threshold) developed on the seventh postoperative day, while the thresholds of the contralateral paws did not change compared to the baseline values. Treatment with the 500 µg·kg^−1^ oral dose of Compound 1, 2, 3 and 4 significantly increased the mechanonociceptive threshold of the treated paw 60 min later showing anti-hyperalgesic effects, while the vehicle had no effect (Compound 1: 52.1 ± 5.4% vs. vehicle: 14.7 ± 6.1%; Compound 2: 54.6 ± 13.6% vs. vehicle: 7.8 ± 8.1%; Compound 3: 57.0 ± 16.1%vs. vehicle: 12.0 ± 7.2%; Compound 4: 57.2 ± 14.6% vs. vehicle: 10.0 ± 7.6%). In case of Compound 2, the 100 µg·kg^−1^ dose also had a significant anti-hyperalgesic effect (Compound 2: 64.4 ± 14.3% vs. vehicle: 7.8 ± 8.1%). Higher doses of the compounds had smaller effects not reaching statistical significance making the dose–response relationship bell-shaped (Figure 5.).

### Spontaneous Locomotor Activity and Anxiety Level Are Not Influenced by Compound 2

Neither spontaneous locomotor activity nor anxiety-related behaviors in the OFT and the EPM were influenced by Compound 2.

**FIGURE 6:**
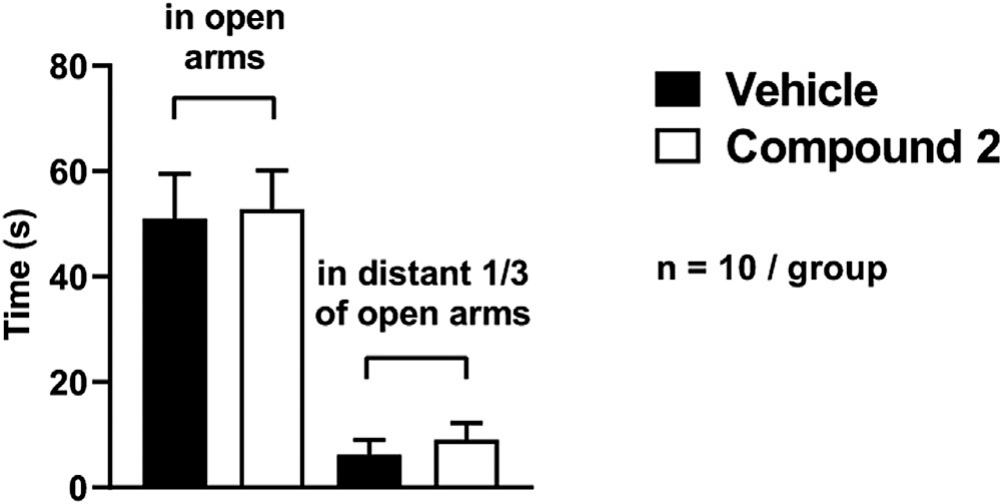
Anxiety-like behavior quantification using EPM. Results are expressed as means ± S.E.M., data were analyzed by Student’s unpaired t‐test, *n* = 10/group.

There was no significant difference in the time spent in the open arms of the EPM (Compound 2: 52.8 ± 7.4 s vs. vehicle: 51.0 ± 8.5 s) or in the distant 1/3 of the open arms (Compound 2: 9.1 ± 3.1 s vs. vehicle: 6.2 ± 2.8) between Compound 2- and vehicle-treated mice (Figure 6.).

**FIGURE 7:**
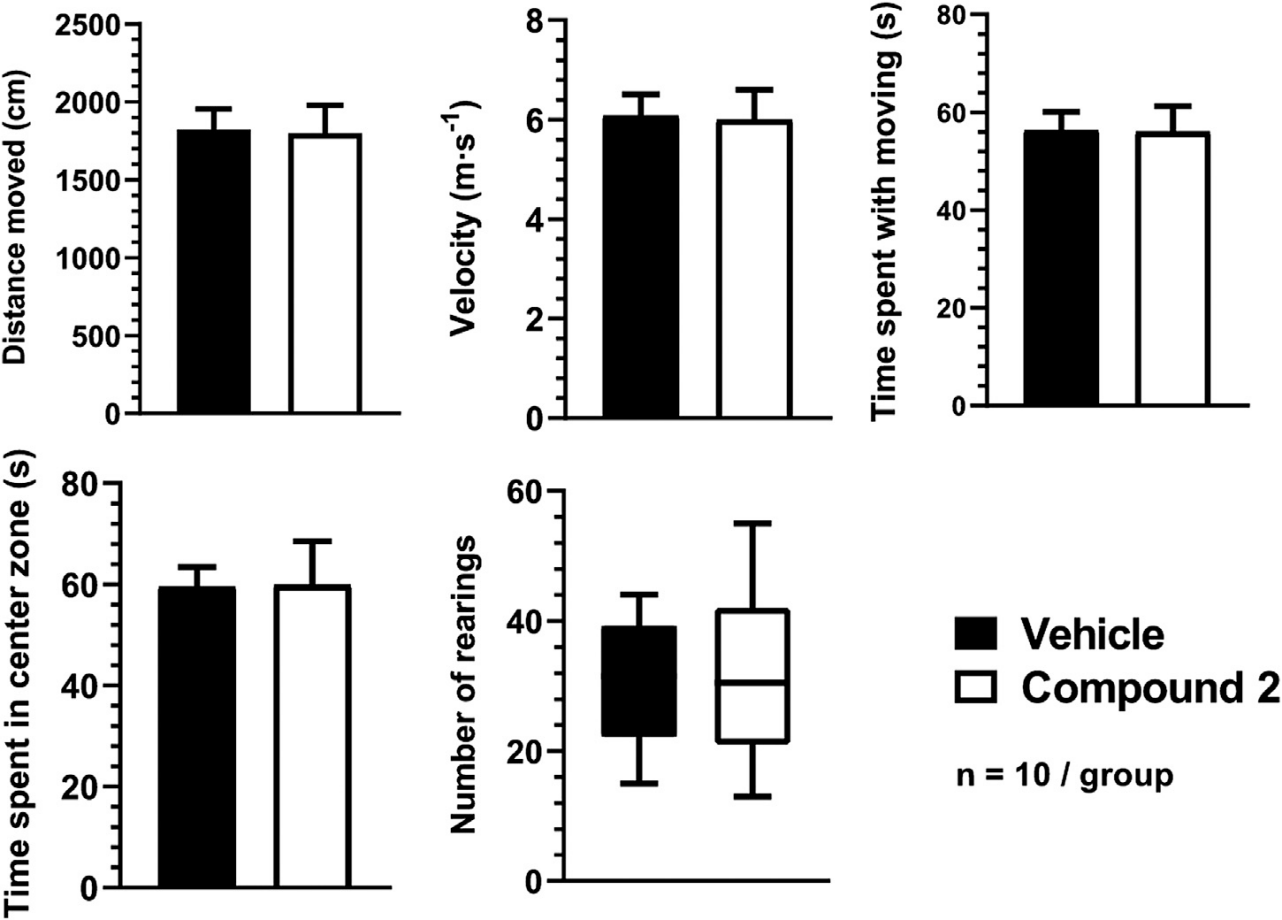
Spontaneous locomotor activity and anxiety-like behavior quantification using OFT. The number of rearings are expressed as the geometric mean with 95% confidence intervals and statistical comparisons were made using the Mann‐Whitney U‐test. All other data are determined as mean ± S.E.M. and were compared using the Student’s unpaired t‐test, *n* = 10/group.

None of the parameters in the OFT, such as the distance moved (Compound 2: 1,798 ± 180.8 cm vs. vehicle: 1,824 ± 130.2 cm), velocity (Compound 2: 6.0 ± 0.6 m/s vs. vehicle: 6.1 ± 0.4 m/s), time spent moving (Compound 2: 56.0 ± 5.2 s vs. vehicle: 56.3 ± 3.7 s), time spent in center zone (Compound 2: 59.8 ± 8.7 s vs. vehicle: 59.5 ± 4.0 s), and number of rearings (Compound 2: 31.1 ± 4.1 vs. vehicle: 30.6 ± 3.2) differed significantly between Compound 2- and vehicle-treated mice (Figure 7.).

## Discussion

In the present study, four novel ligands designed for agonism at SST_4_ somatostatin receptor have been characterized. *In silico* modeling studies revealed that Compounds 1–4 interact with the receptor with similar energy and have overlapping binding sites on the SST_4_ receptor (Liu et al., 2012) as the reference ligands NNC 26-9100 and J-2156 (Table 1). The binding region of J-2156 composed of amino acids Tyr18, Val67, Ser70, Ala71, Cys83, Val259 is overlapped to the binding site called high affinity binding pocket and described by Liu and coworkers (Liu et al., 2012). Docking calculations revealed that Compounds 2–4 maintain the interaction with Asp90 of TM3, as a key residue suggested by previous experimental studies with J-2156 (Kaupmann et al., 1995; Nehrung et al., 1995; Ozenberger and Hadcock, 1995; Chen et al., 1999; Liu et al., 2012). However, neither Compound 1 nor the high affinity reference NNC 26-9100 bind to the conserved aspartic acid. As they have interaction with similar residues in a high percent, our above findings suggest an alternative binding mode for these ligands.

Stimulation of G protein-coupled receptors by agonists regulates multiple downstream pathways through alpha and beta–gamma subunits of the various G proteins. In the G protein activation assay performed on SST_4_ receptor-expressing CHO cells, all the four novel compounds evoked concentration-dependent increases in [^35^S]GTPγS binding reflecting the GDP–GTP exchange reaction on the alpha subunit of G protein similarly to the reference agonists NNC 26-9100 and J-2156. As NNC 26-9100 and J-2156 proved to be full agonists of SST_4_ in a previous study (Liu et al., 1999) and the maximal achievable activation was comparable with that of the other four investigated compounds, all the novel ligands can be considered as full SST_4_ agonists. Based on the EC_50_ values, the novel ligands displayed similar potencies, but Compound 3 was the most potent.

Agonist-evoked activation of heptahelical receptors also stimulates G protein-coupled receptor kinases phosphorylating the activated receptor, thereby allowing attachment of β-arrestin proteins to the receptor. While β-arrestin recruitment/binding physically obstructs the G protein coupling with the receptor, additional mechanisms have been revealed by which β-arrestins ensure efficient blockade of G protein signaling and thus, desensitization of the heptahelical receptors (Shenoy and Lefkowitz, 2011). Although β-arrestins were initially held responsible only for desensitization and down-regulation of these receptors, newer data support the view that they can also initiate several signal transduction mechanisms including e.g. activation of mitogen-activated protein kinase enzymes (Lefkowitz, 2005). Furthermore, the existence of these two distinct pathways (i.e. G protein-dependent and β-arrestin-mediated) allows for biased agonism (also called stimulus trafficking) meaning that some ligands may act exclusively through either G protein-dependent or β-arrestin-mediated cascade (Rajagopal et al., 2010). In case of the SST_4_ receptor, a dissociation of G protein activation and desensitization of a cellular effect with some agonists has been revealed, but the possible role of β-arrestins in the latter response has not been demonstrated so far (Smalley et al., 1998; Engström et al., 2006). In addition, both reference compounds showed an association with β-arrestin 2 recruitment. It was a surprising finding that all four novel SST_4_ receptor agonists failed to evoke any detectable β-arrestin 2 recruitment. This result can be interpreted as biased agonism with Compounds 1–4 meaning that they initiate G_i_ protein-mediated receptor activation, but they are unable to recruit β-arrestin. The latter feature may be advantegous if it results in smaller degree of SST_4_ desensitization to SST_4_ receptor agonists upon repeated administration. However, if β-arrestin-mediated signaling also contributes to some potential therapeutic effects of SST_4_ receptor agonists, this biased agonism may reduce some effects mediated by these receptors. Further studies are needed to clarify these issues. It is worth to mention that the SST_2A_ somatostatin receptor, biased agonism has also been demonstrated (Schonbrunn, 2008).

The high *in vitro* efficacy and potency of the novel SST_4_ agonists made them suitable for *in vivo* testing of their antinociceptive activity. In the mouse model of traumatic neuropathic pain employing partial sciatic nerve ligation (Seltzer et al., 1990), a decrease of the mechanonociceptive threshold of the hindpaw occurred indicating the development of mechanical hyperalgesia. Following oral administration, all novel compounds were able to increase the mechanonociceptive threshold evoking anti-hyperalgesic effects. Interestingly, no conventional dose–response relationship could be established for these drugs. Bell-shaped dose–response curves could be determined for all compounds with two lower and two higher statistically ineffective doses, while the middle dose (500 µg·kg^−1^) produced a significant anti-hyperalgesic effect. Similar efficacies corresponding to about 50–60% anti-hyperalgesic actions were observed for all drugs. Compound 2 also proved to be more potent than the other three ones as it was already effective at the 100 µg·kg^−1^ dose. The (minimal) effective anti-hyperalgesic dose of these novel SST_4_ agonists is rather low indicating high *in vivo* potencies of the compounds. The reason for the bell-shaped dose–response relationship is not clear. The SST_4_ receptors are present in pain-related brain regions (Kecskés et al., 2020) and also on primary sensory neurons including the peripheral terminals. We showed earlier that SST4 activation by the selective agonist J-2156 inhibits the release of sensory neuropeptides, such as substance P, calcitonin gene related peptide and somatostatin (Helyes et al., 2006). Therefore, the potential inhibitory effect of SST_4_ agonists on the release of endogenous inhibitory mediators, such as somatostatin and opioid peptides cannot be excluded and might explain the lack of dose-response relationship or the bell-shaped dose-response curves. The anti-hyperalgesic effect of the compounds is not accompanied by modulated spontaneous locomotor activity and/or anxiety level, as shown by the results obtained with Compound 2, suggesting selective actions on the pain pathway.

We clearly see a significant therapeutic potential in stable, orally active, non-peptide SST_4_ agonists. On the basis of the data obtained with the compounds tested in previous work as well as the present studies, these agents appear to possess broad-spectrum antinociceptive activity in models of both inflammatory and neuropathic pain (Kántás et al., 2019). Regarding the mode of action, a similarity with opioid analgesics is apparent: in both cases G_i_ protein-coupled, typically presynaptically/prejunctionally located receptors are activated. This may result in—among other actions—reduction of the release of a huge array of proinflammatory and/or pronociceptive mediators from peripheral and central endings of nociceptive primary sensory neurons. This mechanism is in sharp contrast with that of receptor antagonists which can only inhibit the action of the endogenous agonist(s) of the respective receptor. As the SST_4_ receptor does not appear to be involved in the myriad of endocrine effects of somatostatin (mediated by SST_2_, SST_3_ and SST_5_ receptors), a good tolerability can be predicted for these agents. A great interest of drug companies is indicated by Lilly’s recently announced licensing agreement for CNTX-0290, a SST_4_ receptor agonist studied in a phase 1 clinical trial (Lilly, 2020; Stevens et al., 2020).

## Conclusion

The novel pyrrolo-pyrimidine compounds are effective and potent SST_4_ receptor agonists as shown by their *in silico* binding to the high affinity binding site and G protein activation on SST_4_-expressing cells, but do not recruit β-arrestin suggesting biased agonism. They inhibit chronic neuropathic mechanical hyperalgesia following a single oral administration of a low dose (500 µg·kg^−1^), therefore, they are promising candidates for the development of a completely novel group of analgesic drugs for a huge unmet medical need.

## Data Availability

The original contributions presented in the study are included in the article/Supplementary Material, further inquiries can be directed to the corresponding author.

## Abbreviations

EF1, elongation factor 1; hsstr_4_, human somatostatin receptor subtype 4; NMRI, naval medical research institute; IRES, internal ribosomal entry site; RLUs, relative luminescence units; RMSD, root mean square deviation; SST_1_-SST_5_, somatostatin receptor subtypes 1–5; TM3, transmembrane domain 3; Tris–EGTA, tris-ethylene glycol bis(2-aminoethyl)tetraacetic acid.
