# Supplementary material for: In Silico, In Vitro and In Vivo Pharmacodynamic Characterization of Novel Analgesic Drug Candidate Somatostatin SST4 Receptor Agonists
Source: Front Pharmacol. 2021 Jan 27;11:601887. doi: 10.3389/fphar.2020.601887 (PMC8015869; doi:10.3389/fphar.2020.601887)
Supplement: Supplementary file 1 [file datasheet1.pdf]

Supplementary Material

Novel pyrrolo-pyrimidine compounds

The preparation route and the synthesized compounds are represented in the Supplementary Scheme 1 and Supplementary Table 1.

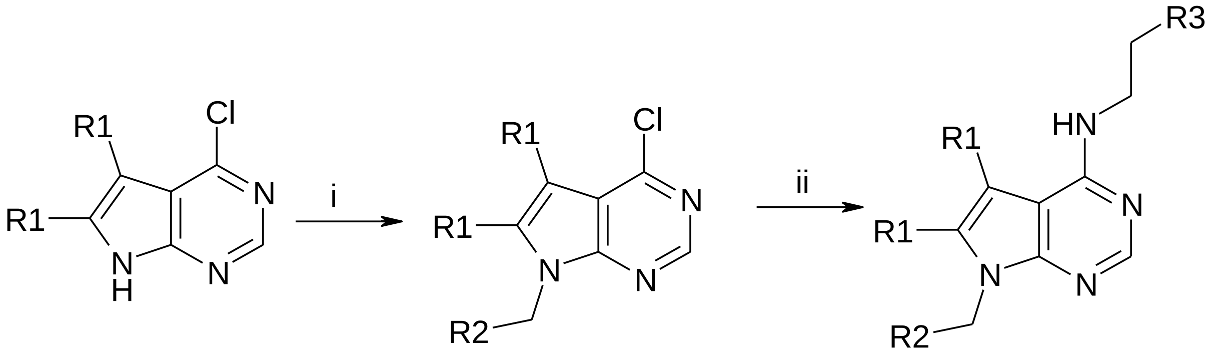

Supplementary Scheme 1 Preparation route. i.) NaH, DMF, $R2CH_2Br$; ii.) R3-ethylamine, DMSO, 100°C.

Supplementary Table 1 Synthesized compounds

	R1	R2	R3
Compound 1	H	phenyl	4-acetamidophenyl
Compound 2	methyl	2-dimethylamino-ethyl	2-chlorophenyl
Compound 3	methyl	phenyl	4-(N',N'diethylureido)-phenyl
Compound 4	methyl	2-furyl	4-acetamidophenyl

Detailed preparation methods of the compounds (Compound 1 = VCC158015, Compound 2 = VCC190907, Compound 3 = VCC808729, Compound 4 = VCC885587)

I. Preparation of [2-(2-Chloro-phenyl)-ethyl]-[7-(3-dimethylamino-propyl)-5,6-dimethyl-7H-pyrrolo[2,3-d]pyrimidin-4-yl]-amine (VCC190907)

1. 4-Chloro-7-(3-dimethylamino-propyl)- 5,6-dimethyl -pyrrolo[2,3-D]pyrimidine

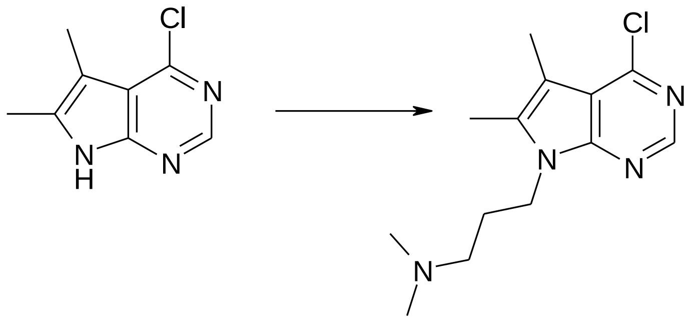

Supplementary Scheme 2

1.82 g (10 mmol) 4-Chloro-5,6-dimethyl-7H-pyrrolo[2,3-D]pyrimidine was solved in 12 ml abs. N,N-dimethylformamide. The solution was cooled down to 0°C, and 480 mg (12 mmol) sodium hydride (60 % dispersion in mineral oil) was added in small amounts. After the addition the reaction mixture was stirred for 30 min at room temperature, then 1.83 g (11 mmol) 3-bromopropyl)dimethylamine was added, and the reaction mixture was stirred overnight. After the starting chlorine compound disappeared by TLC (eluent: chloroform/methanol 10/1) the mixture was diluted with 100 ml ice-cold water. The pH was set to 8-9 with saturated $NaHCO_3$ solution, and the product was extracted with 3x40 ml ethyl acetate. The organic layers were separated, combined, and dried over Na_2SO_4. The solvent was removed under vacuum, the remaining oil was treated with diisopropyl-ether to obtain the solid product which was used for the next step without further purification.

Yield: 2.05 g (77 %) yellow material.

2. [2-(2-Chloro-phenyl)-ethyl]-[7-(3-dimethylamino-propyl)-5,6-dimethyl-7H-pyrrolo[2,3-d]pyrimidin-4-yl]-amine (VCC190907)

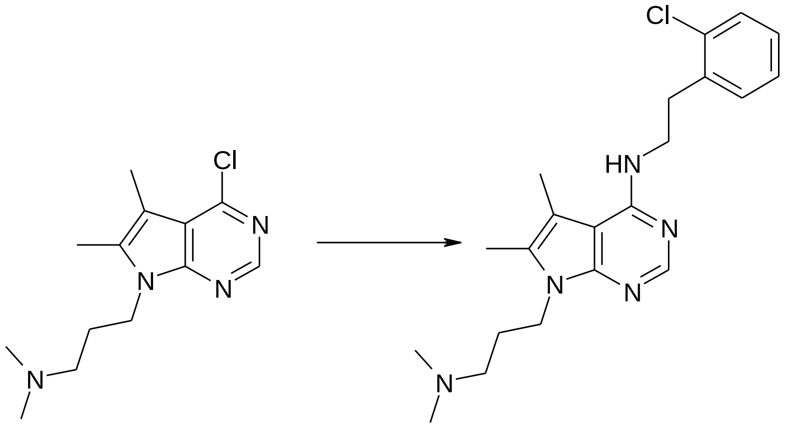

Supplementary Scheme 3

1.33 g (5 mmol) 4-Chloro-7-(3-dimethylamino-propyl)-5,6-dimethyl-pyrrolo[2,3-D]pyrimidine (obtained from the previous step), and 1.556 g (10 mmol) 2-(2-Chlorophenyl)ethylamine were solved in 6 ml dimethylsulfoxide, and the mixture was stirred at 100°C for 12 hours. The reaction mixture was cooled down to room temperature, then it was diluted with 120 ml saturated NaHCO3 solution. The product was extracted with 3x40 ml ethyl acetate. The organic layers were separated, combined, and dried over Na_2SO_4. The solvent was removed under vacuum, the crude product was purified by column chromatography (eluent: chloroform/methanol 10/1, with 1% NH_3.aq). The yielded oil was dissolved in 30 ml diethyl ether, and 5 ml saturated HCl/ethyl acetate was added. The formed HCl salt was filtered out, and dried.

Yield: 1.33 g (63 %) off-white crystals.

Melting point: 245-248°C.

LCMS: 99 % (Rt: 2.36 min)

1H-NMR: 10.78(bs, 1H), 8.27(s, 1H), 7.94(bs, 1H), 7.48(d, 1H), 7.42(d, 1H), 7.28(m, 2H), 4.26(t, 2H), 3.91(q, 2H), 3.11(t, 2H), 3.04(bs, 2H), 2.70(s, 6H), 2.36(s, 3H), 2.39(s, 3H), 2.09(m, 2H)

II. Preparation of 3-{4-[2-(7-Benzyl-5,6-dimethyl-7H-pyrrolo[2,3-d]pyrimidin-4-ylamino)-ethyl]-phenyl}-1,1-diethyl-urea (VCC808729)

1. 4-Chloro-7-benzyl- 5,6-dimethyl-pyrrolo[2,3-D]pyrimidine

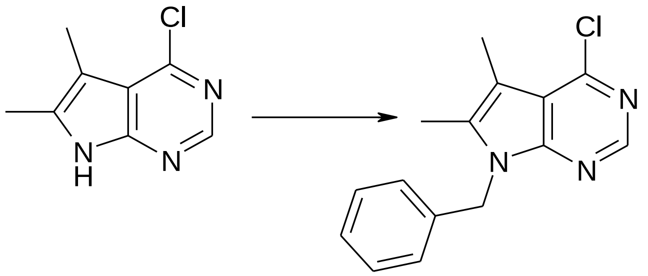

Supplementary Scheme 4

1.456 g (8 mmol) 4-Chloro-5,6-dimethyl-7H-pyrrolo[2,3-D]pyrimidine was solved in 10 ml abs. N,N-dimethylformamide. The solution was cooled down to 0°C, and 385 mg (9.6 mmol) sodium hydride (60 % dispersion in mineral oil) was added in small amounts. After the addition the reaction mixture was stirred for 30 min at room temperature, then 1.88 g (11 mmol) benzyl bromide was added and stirred overnight. After the starting chlorine compound disappeared by TLC (eluent: chloroform/methanol 10/1) the mixture was diluted with 100 ml ice-cold water. The pH was set to 8-9 with saturated $NaHCO_3$ solution, and the product was extracted with 3x40 ml ethyl acetate. The organic layers were separated, combined, and dried over Na_2SO_4. The solvent was removed under vacuum, the remaining oil was treated with diisopropyl-ether to obtain the solid product which was used for the next step without further purification.

Yield: 1.76 g (81 %) off-white material.

2. 3-{4-[2-(7-Benzyl-5,6-dimethyl-7H-pyrrolo[2,3-d]pyrimidin-4-ylamino)-ethyl]-phenyl}-1,1-diethyl-urea (VCC808729)

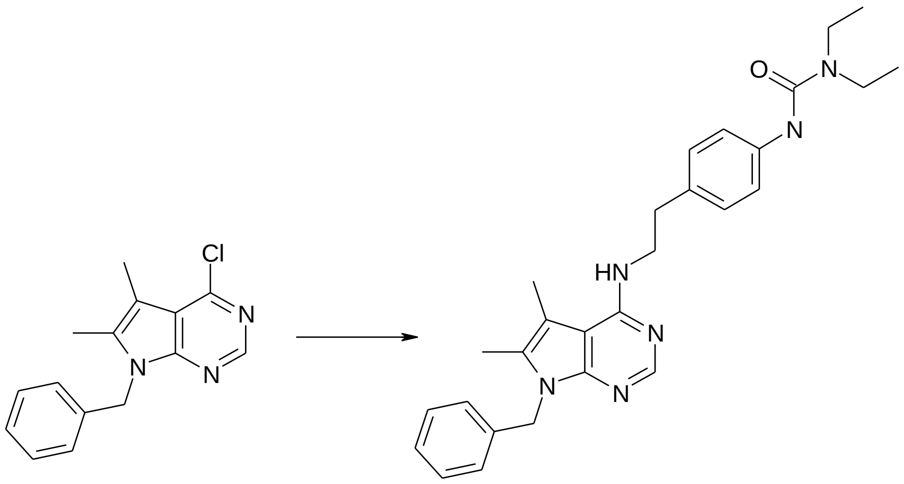

Supplementary Scheme 5

1.63 g (6 mmol) 4-Chloro-7-benzyl- 5,6-dimethyl-pyrrolo[2,3-D]pyrimidine (obtained from the previous step), and 1.88 g (8 mmol) 4-(2-aminoethyl)-phenyl-1,1-diethyl-urea were solved in 8 ml dimethylsulfoxide, and the mixture was stirred at 100°C for 12 hours. The reaction mixture was cooled down to room temperature, then it was diluted with 120 ml saturated $NaHCO_3$ solution. The product was extracted with 4x40 ml ethyl acetate. The organic layers were separated, combined, and dried over Na_2SO_4. The solvent was removed under vacuum, the crude product was purified by column chromatography (eluent: chloroform/methanol 10/1, with 1 % NH_3.aq).

Yield: 1.47 g (52 %) off-white crystals.

Melting point: 138.5-139°C

LCMS: 99 % (3.36, 3.68 min)

1H-NMR: 8.10 (s, 1H), 8.05 (s, 1H), 7.41 (dm, J = 7.5 Hz, 2H), 7.16-7.36 (ovl. m, 3H), 7.12 (dm, J = 7.5 Hz, 2H), 7.03 (dm, J = 7.0 Hz, 2H), 6.35 (t, J ~ 6 Hz, 1H), 5.34 (s, 2H), 3.67 (q, J ~ 7.0 Hz, 2H), 3.31 (q, J = 6.8 Hz, 4H), 2.85 (t, J ~ 7.0 Hz, 2H)

III. Preparation of N-{4-[2-(7-Furan-2-ylmethyl-5,6-dimethyl-7H-pyrrolo[2,3-d]pyrimidin-4-ylamino)-ethyl]-phenyl}-acetamide (VCC885587)

1. 4-Chloro-7-(furan-2-ylmethyl)- 5,6-dimethyl-pyrrolo[2,3-D]pyrimidine

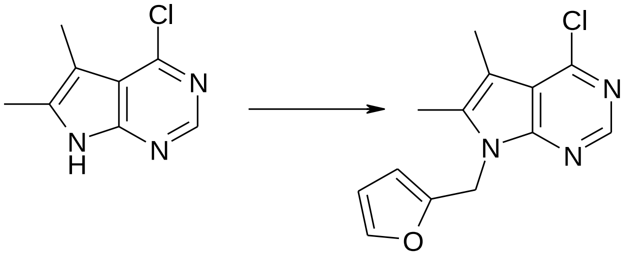

Supplementary Scheme 6

1.456 g (8 mmol) 4-Chloro-5,6-dimethyl-7H-pyrrolo[2,3-D]pyrimidine was solved in 10 ml abs. N,N-dimethylformamide. The solution was cooled down to 0°C, and 385 mg (9.6 mmol) sodium hydride (60 % dispersion in mineral oil) was added in small amounts. After the addition the mixture was stirred for 30 min at room temperature, then 1.77 g (11 mmol) 2-(bromomethyl)furan was added, and the mixture was stirred for 24 hours. After the starting chlorine compound disappeared by TLC (eluent: chloroform/methanol 10/1) the mixture was diluted with 100 ml ice-cold water. The pH was set to 8-9 with saturated $NaHCO_3$ solution, and the product was extracted with 3x40 ml ethyl acetate. The organic layers were separated, combined, and dried over Na_2SO_4. The solvent was removed under vacuum, the remaining oil was treated with diisopropyl-ether to obtain the solid product which was used for the next step without further purification.

Yield: 1.80 g (86 %) yellow material.

2. N-{4-[2-(7-Furan-2-ylmethyl-5,6-dimethyl-7H-pyrrolo[2,3-d]pyrimidin-4-ylamino)-ethyl]-phenyl}-acetamide (VCC885587)

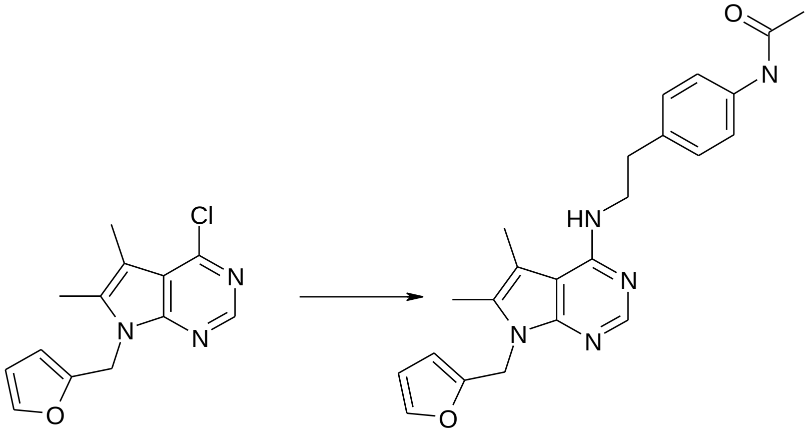

Supplementary Scheme 7

1.80 g (6.88 mmol) 4-Chloro-7-benzyl- 5,6-dimethyl-pyrrolo[2,3-D]pyrimidine (obtained from the previous step), and 1.60 g (9 mmol) N-[4-(2-Aminoethyl)phenyl]acetamide were solved in 10 ml dimethylsulfoxide, and the mixture was stirred at 100°C for 16 hours. The reaction mixture was cooled down to room temperature, then it was diluted with 140 ml saturated $NaHCO_3$ solution. The product was extracted with 5x50 ml ethyl acetate. The organic layers were separated, combined, and dried over Na_2SO_4. The solvent was removed under vacuum, the crude product was purified by column chromatography (eluent: chloroform/methanol 10/1).

Yield: 1.69 g (61 %) yellowish crystalline material.

Melting point: 206.8-208.1°C

LCMS: 100 % (Rt: 2.76 min)

1H-NMR: 9.88(bs, 1H), 8.10(s, 1H), 7.52(s, 1H), 7.00(d, 2H), 7.16(d, 2H), 6.36(d, 1H), 6.33(t, 1H), 6.23(d, 1H) 5.30(s, 2H), 3.66(q, 2H), 2.85(t, 2H), 2.26(s, 3H), 2.02(s, 3H)

IV. Preparation of N-{4-[2-(7-Benzyl-7H-pyrrolo[2,3-d]pyrimidin-4-ylamino)-ethyl]-phenyl}-acetamide (VCC158015)

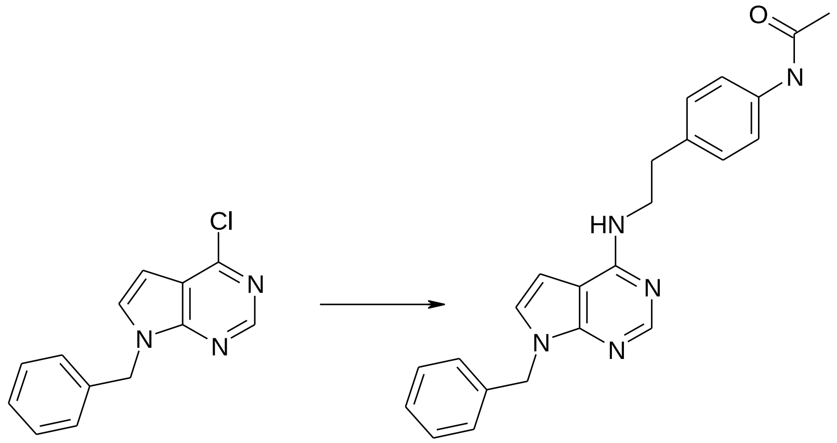

Supplementary Scheme 8

1.00 g (4.10 mmol) 7-Benzyl-4-chloro-7H-pyrrolo[2,3-D] pyrimidine (purchased commercially), and 950 mg (5.33 mmol) N-[4-(2-Aminoethyl)phenyl]acetamide were solved in 4 ml

dimethylsulfoxide, and the mixture was stirred at 100°C for 16 hours. The reaction mixture was cooled down to room temperature, then it was diluted with 80 ml saturated $NaHCO_3$ solution. The product was extracted with 4x25 ml ethyl acetate. The organic layers were separated, combined, and dried over Na_2SO_4. The solvent was removed under vacuum, the crude product was purified by column chromatography (eluent: chloroform/methanol 10/1).

Yield: 1.14 g (72 %)

LCMS: 97 % (Rt: 2.73, 3.06 min)

1H-NMR: 9.84(bs.1H), 8.16(s,1H), 7.57(t,1H), 7.48(d,2H), 7.33-7.16(m 8H), 6.58(d,1H), 5.33(s,2H), 3.65(q,2H), 2.85(t,2H), 2.01(s,3H)

Changes of the mechanonociceptive thresholds

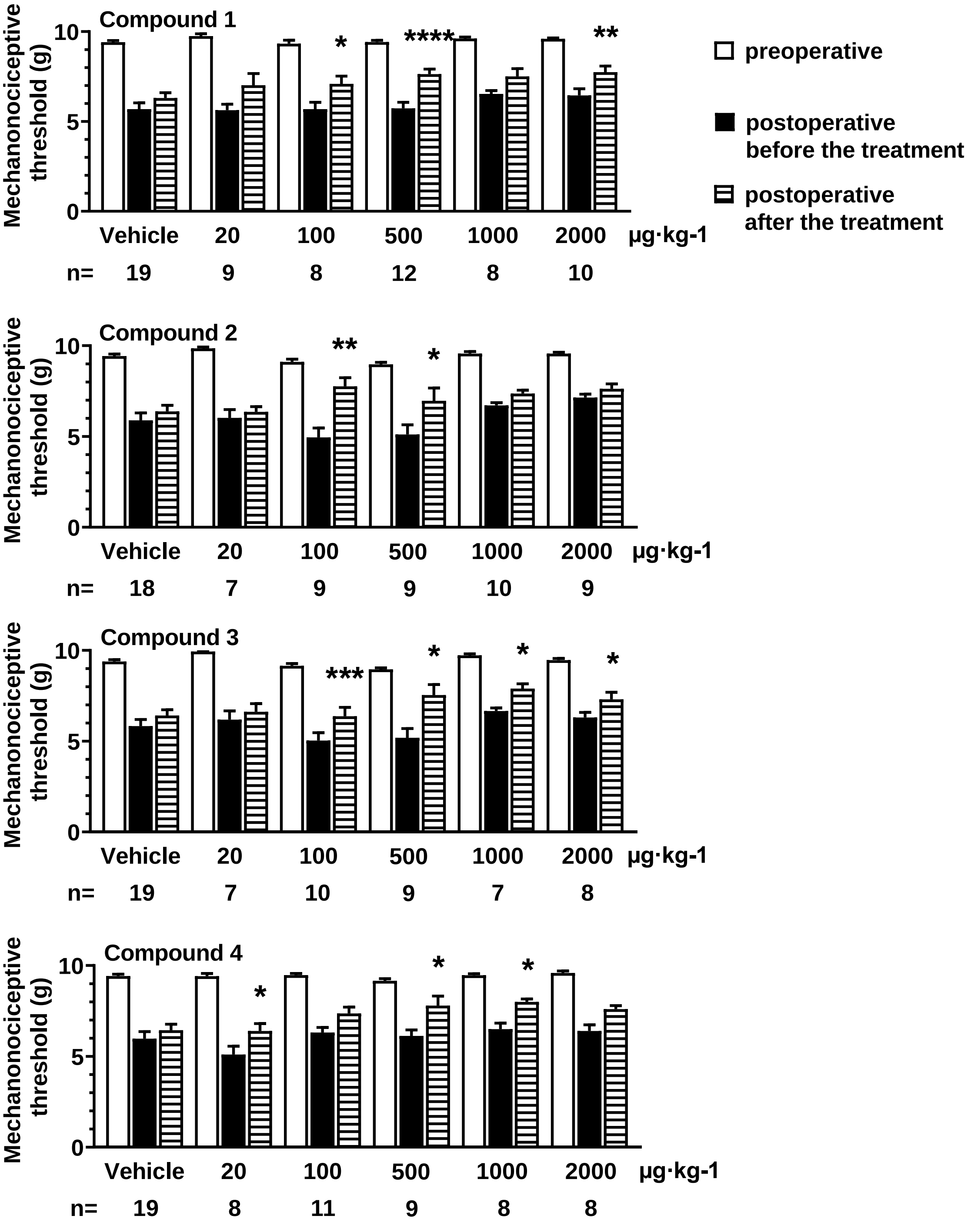

Supplementary Figure 1

Changes of the mechanonociceptive thresholds. Columns represent the mechanonociceptive thresholds before the operation (white) and on the 7th postoperative day before (black) and 60 min after the treatment with Compounds 1–4 (striped). Each column represents the mean+S.E.M. of n. Data were analysed with two-way ANOVA Bonferroni's Multiple Comparison Test (*p<0.05, **p<0.01, ***p<0.001, ****<0.0001 vs. postoperative values before the treatment).

Supplementary Table 1

**Compound 1**	**Vehicle**	**20 µg/kg**	**100 µg/kg**	**500 µg/kg**	**1000 µg/kg**	**2000 µg/kg**
**preoperative (g)**	9.4±0.1	9.8±0.1	9.3±0.2	9.4±0.1	9.6±0.1	9.6±0.1
**postoperative before the treatment (g)**	5.7±0.3	5.6±0.3	5.7±0.4	5.7±0.3	6.5±0.2	6.5±0.4
**postoperative after the treatment (g)**	6.3±0.3	7.0±0.7	7.1±0.4	7.6±0.3	7.5±0.4	7.7±0.3
**Compound 2**	**Vehicle**	**20 µg/kg**	**100 µg/kg**	**500 µg/kg**	**1000 µg/kg**	**2000 µg/kg**
**preoperative (g)**	9.4±0.1	9.9±0.1	9.1±0.1	9.0±0.1	9.6±0.1	9.6±0.1
**postoperative before the treatment (g)**	5.9±0.4	6.0±0.5	0.4±0.5	5.1±0.5	6.7±0.2	7.1±0.2
**postoperative after the treatment (g)**	6.4±03	6.4±0.3	7.8±0.5	7.0±0.7	7.4±0.2	7.6±0.3
**Compound 3**	**Vehicle**	**20 µg/kg**	**100 µg/kg**	**500 µg/kg**	**1000 µg/kg**	**2000 µg/kg**
**preoperative (g)**	9.4±0.1	9.9±0.1	9.2±0.1	9.0±0.1	9.7±0.1	9.5±0.1
**postoperative before the treatment (g)**	5.8±0.4	6.2±0.5	5.0±0.4	5.2±0.5	6.7±0.2	6.3±0.3

**postoperative after the treatment (g)**	6.4±0.3	6.6±0.4	6.4±0.5	7.5±0.6	7.9±0.3	7.3±0.4
**Compound 4**	**Vehicle**	**20 µg/kg**	**100 µg/kg**	**500 µg/kg**	**1000 µg/kg**	**2000 µg/kg**
**preoperative (g)**	9.4±0.1	9.5±0.2	9.5±0.1	9.2±0.1	9.5±0.1	9.6±0.1
**postoperative before the treatment (g)**	6.0±0.4	5.1±0.5	6.3±0.3	6.1±0.3	6.5±0.3	6.4±0.3
**postoperative after the treatment (g)**	6.4±0.3	6.4±0.4	7.4±0.3	7.8±0.5	8.0±0.2	7.6±0.2

Mechanonociceptive thresholds on the ipsilateral hindpaws in grams. Data are expressed as means±S.E.M and visualized in Supplementary Figure1.
